# Joint association of socioeconomic circumstances and minor mental health problems with antidepressant medication

**DOI:** 10.1093/eurpub/ckac048

**Published:** 2022-06-03

**Authors:** Aino Salonsalmi, Elina Mauramo, Ossi Rahkonen, Olli Pietiläinen, Eero Lahelma

**Affiliations:** Department of Public Health, University of Helsinki, Helsinki, Finland; Department of Public Health, University of Helsinki, Helsinki, Finland; Department of Public Health, University of Helsinki, Helsinki, Finland; Department of Public Health, University of Helsinki, Helsinki, Finland; Department of Public Health, University of Helsinki, Helsinki, Finland

## Abstract

**Background:**

Disadvantageous socioeconomic circumstances and minor mental health problems have both been associated with mental disorders, such as depression, but their joint contribution remains unknown.

**Methods:**

The Helsinki Health Study baseline survey (2000–02) of 40- to 60-year-old employees was linked with antidepressant medication data from registers of the Social Insurance Institution of Finland. The analyses were made using logistic regression with first prescribed antidepressant medication purchase during a 10-year follow-up as the outcome. Minor mental health problems were measured by the emotional well-being scale of the RAND-36. Odds ratios were calculated for joint association of the lowest quartile of the emotional well-being scale of the RAND-36 and socioeconomic circumstances. Childhood (parental education and childhood economic difficulties), conventional (education, occupational class and income) and material (housing tenure and current economic difficulties) socioeconomic circumstances were examined. This study included 5450 participants.

**Results:**

Minor mental health problems dominated the joint associations. Minor mental health problems were associated with antidepressant medication irrespective of socioeconomic circumstances whereas only low income, current economic difficulties and living in rented housing showed an association without minor mental health problems at baseline. Marital status, working conditions and BMI and health behaviours had only minimal contributions to the associations.

**Conclusions:**

Minor mental health problems were consistently and strongly associated with antidepressant medication and dominated the joint associations with socioeconomic circumstances. Paying attention to minor mental health problems might help prevent mental disorders such as depression.

## Introduction

Mental disorders increasingly contribute to the global burden of disease and their importance has expanded in the past decades.^1^ Depression is the most prevalent mental disorder and one of the leading causes of disability worldwide.^2^ The incidence of mental disorders has risen in Finland during the past decades. It is estimated that in Finland 5–7% of population suffer from major depression during each year.^3^^,^^4^ In working life, mental disorders are the most common reason behind work disability with depression being the leading mental disorder behind disability retirement in Finland.^5^

In previous research, socioeconomic differences in major depression have followed the pattern of somatic diseases with people in lower socioeconomic positions suffering disproportionally from poorer health. A meta-analysis concluded that disadvantageous socioeconomic circumstances were associated with the onset of depression and increased the risk of persistent depression.^6^ More recent studies have supported the findings.^7–9^ In contrast to major depression and other severe mental health problems socioeconomic differences in less severe minor mental health problems, such as symptoms of depression and anxiety lack a clear gradient.^10–12^ Previous studies have suggested a prevalence of minor mental health problems up to 30% among employees.^13^

Both disadvantageous socioeconomic circumstances^6^ and minor mental health problems^14–16^ have been associated with major depression. Their joint contribution, however, remains unknown. Disadvantageous socioeconomic circumstances might additionally burden individuals with minor mental health problems, increasing their risk of major depression. Advantageous socioeconomic circumstances might in turn protect from minor mental health problems developing into major depression. In addition, a mismatch between the need and receipt of treatment^17^ might widen the gap further. A previous Finnish study among public sector employees found that men in low socioeconomic positions were less likely to have antidepressant medication although they faced an increased risk of mental health-related mortality.^17^ Purchases of prescribed antidepressant medication offer a register-based measure of depression not affected by self-report bias. In addition to the presence of mental disorders, having medication reflects seeing a medical doctor and receiving medical treatment. Those with advantageous socioeconomic circumstances might be more prone to seek treatment and thus receive antidepressant medication.

Conventional socioeconomic measures namely education, occupational class and income have been associated with antidepressant medication^18^^,^^19^ in some but not all studies.^17^^,^^20^^,^^21^ Socioeconomic differences in health are, however, a product of socioeconomic differences in various resources, health-endangering exposures and health-protecting factors which act throughout the life course. Thus, the conventional measures of adult socioeconomic circumstances are unlikely to fully cover the different domains of socioeconomic measures. The associations between further socioeconomic circumstances and antidepressant medication have been less studied but a Swedish study reported that low household income, difficulties in paying bills and lack of cash reserves were associated with an increased risk of antidepressant medication.^22^ Studies have also suggested that childhood economic difficulties^18^^,^^23^ and low parental education^24^ increase the risk of psychotropic medication.

We aimed to examine the joint associations of past and present socioeconomic circumstances and minor mental health problems with antidepressant medication among midlife and ageing employees. We used a multiple framework of socioeconomic circumstances by including childhood socioeconomic circumstances namely parental education and childhood economic difficulties, conventional measures of socioeconomic circumstances namely education, occupational class and income and material circumstances namely housing tenure and current economic difficulties.

## Methods

This study is part of the Helsinki Health Study (HHS) among the employees of the City of Helsinki,^25^ the largest employer in Finland with about 37 000 employees.^26^ The jobs include white- and blue-collar jobs, such as teachers, lawyers, nurses, doctors, bus drivers, child minders and garden workers. The majority of the employees (76%) are women corresponding to the Finnish municipal sector.

Data on socioeconomic circumstances, minor mental health problems and covariates were derived from the HHS baseline mail surveys conducted in 2000, 2001 and 2002 among employees who turned 40, 45, 50, 55 or 60 during those years. The target population was 13 346 and 8960 participated with a response rate of 67%. Data on prescribed reimbursed antidepressant medication purchases came from the registers of the Social Insurance Institution of Finland and were linked with the survey data among participants (*n* = 6603, 74% of the participants) who consented to the linkage. Purchases of prescribed antidepressant medication were followed from the day of returning the baseline questionnaire until 10 years, until the date of antidepressant medication purchase or until death (*n* = 84). Participants with antidepressant medication purchases during the 3 years preceding the baseline survey were excluded (*n* = 751). After exclusions due to missing data on minor mental health problems or covariates (*n* = 373) the study included 5450 employees of whom 4211 were women and 1239 men. There was item-non-response on measures of socioeconomic circumstances and the final numbers in individual analyses (table 1) were slightly smaller.

**Table 1:** Distributions of socioeconomic circumstances, minor mental health problems and antidepressant medication purchases. Cumulative incidence of antidepressant medication by socioeconomic circumstances and minor mental health problems (%)

	*n*	%	% with antidepressant medication during follow-up
Parental education			
High	1244	23	22
Low	4172	77	20
*P*-value			0.287
Childhood economic difficulties			
No	4199	83	20
Yes	858	17	25
*P*-value			0.001
Education			
High	3345	62	21
Low	2078	38	21
*P*-value			0.808
Occupational class			
High	2868	53	20
Low	2577	47	22
*P*-value			0.235
Household income			
High	2735	51	18
Low	2612	49	23
*P*-value			<0.001
Current economic difficulties			
No	2961	54	18
Yes	2475	46	24
*P*-value			<0.001
Housing tenure			
Owner	3703	68	19
Renter	1718	32	26
*P*-value			<0.001
Minor mental health problems			
No	4002	73	16
Yes	1448	27	34
*P*-value			<0.001
Antidepressant medication purchase during follow-up			
No	4317	79	–
Yes	1133	21	–

The non-response analysis of the HHS found that the response rate tended to be lower for employees who were younger, in lower occupational classes and with increased sickness absence during the survey year. These differences were minor and not fully consistent. There were only small differences in consenting to the data linkages but men gave their consent more often than women.^25^^,^^27^

The Ethics Committee of the Department of Public Health at the University of Helsinki and the health authorities at the City of Helsinki approved the study.

### Socioeconomic circumstances

Parental education was based on either maternal or paternal education whichever was higher, and was divided into ‘low’ (elementary school or part of it, intermediate school and vocational school) and ‘high’ (matriculation examination, college-level training, polytechnic or university degree). Childhood economic difficulties were measured by asking whether there were economic difficulties in the family before the respondent turned 16 (yes/no). Respondent’s own education was divided into ‘low’ (elementary school, intermediate school and vocational school) and ‘high’ (matriculation examination, college-level training, polytechnic or university degree). Occupational class was based on the job title and divided into ‘low’ (non-manual employees, such as clerical employees and child minders and manual workers, such as cleaning workers) and ‘high’ (managers and professionals, such as teachers and physicians and semi-professionals, such as nurses and foremen). Monthly household income was dichotomized by the median into ‘low’ and ‘high’. Housing tenure was divided into owner-occupiers and renters. Current economic difficulties were measured by asking (i) how often the respondent had enough money to buy clothing and food needed by the family, and (ii) how much the respondent had difficulties in paying bills. A combined variable was formed: ‘No difficulties’ and ‘difficulties’ the latter including both occasional and frequent difficulties.

### Minor mental health problems

Minor mental health problems were measured by the emotional well-being scale of the RAND-36.^28^ The RAND-36 is a reliable and well-validated self-report health survey instrument developed in the Medical Outcomes Study. Emotional well-being scale was assessed by five items indicating emotional well-being, which inquired how much of the time during the past 4 weeks the respondent had been very nervous, had felt so down that nothing could cheer him/her up, had felt calm and peaceful, had felt downhearted and blue and had been a happy person. The score ranges from 0 to 100 and was divided into quartiles. Individuals in the lowest quartile were classified as having minor mental health problems.

### Antidepressant medication

The outcome measure of the study was the first purchase of prescribed, reimbursed antidepressant medication. Medication purchases were classified according to the Anatomical Therapeutic Chemical system by WHO.^29^ Antidepressant medication consisted of the group N06A. There were 1133 events during the follow-up.

### Covariates

Age and gender were included as covariates. Marital status was divided into ‘single’, ‘married or cohabiting’ and ‘divorced or widowed’. There was a single-item question inquiring how mentally strenuous the respondent considered the work with response alternatives ranging from ‘very light’ to ‘very heavy’. A similar question inquired physical strenuousness of the work. The four response alternatives were reduced to three groups. Smoking was divided into smokers and non-smokers. Body mass index (BMI) was calculated from self-reported weight and height and divided into under 25, between 25 and 30 and above 30 kg/m^2^. Problem drinking was measured by the CAGE-scale (cutting down, annoyed by criticism, guilty, eye-opener).^30^ Leisure-time physical activity was measured by four questions from which metabolic equivalent tasks were calculated and included as a continuous variable.

### Statistical methods

Differences in the distributions of antidepressant medication purchases by socioeconomic variables and mental health problems were tested by the chi-squared test. The associations between socioeconomic circumstances, minor mental health problems and antidepressant medication were further analyzed by logistic regression analysis yielding odds ratios (OR) and their 95% confidence intervals (95% CIs). The first reimbursed antidepressant medication purchase during a 10-year follow-up served as outcome variable. The SAS statistical program version 9.4 was used in performing the analyses (SAS Institute Inc., Cary, NC, USA).

First, we fitted models separately for the associations between socioeconomic circumstances and antidepressant medication and between minor mental health problems and antidepressant medication. Interactions for gender were tested and statistically significant interaction (*P* = 0.0251) was found for the association between education and antidepressant medication. This association was presented separately for men and women but otherwise the analyses were made on pooled data.

In the joint models, participants in advantageous socioeconomic circumstances and without minor mental health problem served as reference categories. First, base models adjusted for age and gender were fitted. Next, other covariates were added to the base models one by one: first marital status, next working conditions and finally BMI and health behaviours. As there were no interactions for gender regarding the joint models, men and women were pooled in the analyses. Only those socioeconomic circumstances that were themselves associated with antidepressant medication were included in the joint models. Finally, we fitted joint models adjusted simultaneously for all socioeconomic circumstances that were themselves associated with antidepressant medication. As item-response concerning socioeconomic circumstances only partially overlapped new base models including age and gender were also fitted among 4942 participants with full data on covariates and all socioeconomic circumstances.

To examine the synergistic interactions between socioeconomic circumstances and minor mental health problems the synergy indices (*S*) were calculated using the following equation: *S* = OR (socioeconomic exposure with minor mental health problems − 1)/[(OR no socioeconomic exposure with minor mental health problems − 1) + (OR socioeconomic exposure without minor mental health problems − 1)]. A synergy index above 1 suggests that the joint association is synergistic, a synergy index =1 suggests an additive association and a synergy index below 1 an antagonistic association.^31^

## Results

Low education was the most common parental education type (77%) whereas the majority (83%) had not experienced any childhood economic difficulties (table 1). High own education was more common than low one whereas occupational class was distributed rather evenly. Current economic difficulties were common (46%) and the majority owned their housing (68%). During the follow-up 20% of the participants had an antidepressant medication purchase and these varied by socioeconomic circumstances and minor mental health problems (Supplementary table S1). Employees with minor mental health problems had medication purchases more often than employees without them.

The age- and gender-adjusted associations of socioeconomic circumstances and minor mental health problems with antidepressant medication in table 2 shows that there was an association between childhood economic difficulties and antidepressant medication (OR 1.38, 95% CI 1.16–1.65) (table 2). Of the conventional measures of socioeconomic circumstances, low education and low occupational class were unassociated with antidepressant medication whereas low income showed an association (OR 1.37, 95% CI 1.20–1.57). Current economic difficulties (OR 1.37, 95% CI 1.20–1.56) and living in rented housing (OR 1.45, 95% CI 1.27–1.67) were both associated with antidepressant medication. Minor mental health problems showed the strongest association with antidepressant medication (OR 2.66, 95% CI 2.31–3.05).

**Table 2:** The associations between socioeconomic circumstances and antidepressant medication and the association between minor mental health problems and antidepressant medication (ORs and their 95% CIs)

	The models were adjusted for age and gender
Parental education	
High	1.00
Low	0.92 (0.79–1.07)
Childhood economic difficulties	
No	1.00
Yes	1.38 (1.16–1.65)
Education	
** **Women	
High	1.00
Low	1.13 (0.97–1.31)
** **Men	
High	1.00
Low	0.81 (0.59–1.12)
Occupational class	
High	1.00
Low	1.04 (0.91–1.19)
Household income	
High	1.00
Low	1.37 (1.20–1.57)
Current economic difficulties	
No	1.00
Yes	1.37 (1.20–1.56)
Housing tenure	
Owner	1.00
Renter	1.45 (1.27–1.67)
Minor mental health problems	
No	1.00
Yes	2.66 (2.31–3.05)

The joint associations of socioeconomic circumstances and minor mental health problems with antidepressant medication are presented in table 3. Socioeconomic circumstances not themselves associated with antidepressant medication were not included in the joint models. There was a strong association with antidepressant medication among individuals with minor mental health problems both with (OR 3.06, 95% CI 2.39–3.93) and without (OR 2.70, 95% CI 2.29–3.18) childhood economic difficulties. Adjustments for marital status, working conditions, BMI and health had only minimal contributions. Low household income was weakly associated with antidepressant medication without minor mental health problems (OR 1.21, 95% CI 1.02–1.44). Individuals with minor mental health problems showed a strong association with increased antidepressant medication and the association was strongest for individuals with low income (OR 3.45, 95% CI 2.85–4.18). Adjustments for BMI and health behaviours minimally attenuated the associations. Current economic difficulties without minor mental health problems were associated with increased antidepressant medication (OR 1.35, 95% CI 1.14–1.60). Individuals without current economic difficulties and with minor mental health problems showed a strong association (OR 2.96, 95% CI 2.41–3.63). The association was equally strong for individuals with both current economic difficulties and minor mental health problems (OR 3.09, 95% CI 2.56–3.73). Adjustments for marital status and working conditions had negligible effect. Adjustment for BMI and health behaviours minimally attenuated the association. Living in rented housing without minor mental health problems was associated with increased antidepressant medication (OR 1.48, 95% CI 1.24–1.76). Individuals with minor mental health problems showed a strong association with increased antidepressant medication irrespective of housing tenure (OR 2.72, 95% CI 2.28–3.24 for owners; OR 3.69, 95% CI 2.98–4.57 for renters). Adjustments for marital status and for working conditions had no contribution. Adjustments for weight and health behaviours slightly attenuated the associations. Synergy index suggested a synergistic association regarding household income (1.54) and housing tenure (*S* = 1.22).

**Table 3:** The joint association of socioeconomic circumstances and minor mental health problems with antidepressant medication (ORs and their 95% CIs)

	Gender, age= Model 1	Model 1 + marital status	**Model 1 + working conditions** ^a^	**Model 1 + weight and health behaviours** ^b^
Childhood economic difficulties				
No, no minor mental health problem	1.00	1.00	1.00	1.00
Yes, no minor mental health problem	1.25 (0.99–1.60)	1.24 (0.98–1.58)	1.25 (0.98–1.59)	1.20 (0.94–1.53)
No, minor mental health problem	2.70 (2.29–3.18)	2.66 (2.25–3.13)	2.63 (2.23–3.11)	2.53 (2.14–3.00)
Yes, minor mental health problem	3.06 (2.39–3.93)	2.98 (2.32–3.82)	2.97 (2.31–3.82)	2.63 (2.04–3.40)
Synergy index	1.06			
Household income				
High, no minor mental health problem	1.00	1.00	1.00	1.00
Low, no minor mental health problem	1.21 (1.02–1.44)	1.16 (0.96–1.41)	1.26 (1.06–1.50)	1.18 (0.99–1.40)
High, minor mental health problem	2.38 (1.93–2.93)	2.37 (1.92–2.91)	2.27 (1.84–2.81)	2.18 (1.77–2.70)
Low, minor mental health problem	3.45 (2.85–4.18)	3.30 (2.67–4.07)	3.44 (2.84–4.18)	3.11 (2.56–3.77)
Synergy index	1.54			
Current economic difficulties				
No, no minor mental health problem	1.00	1.00	1.00	1.00
Yes, no minor mental health problem	1.35 (1.14–1.60)	1.31 (1.10–1.56)	1.37 (1.15–1.62)	1.29 (1.08–1.53)
No, minor mental health problem	2.96 (2.41–3.63)	2.94 (2.40–3.61)	2.85 (2.32–3.50)	2.87 (2.26–3.42)
Yes, minor mental health problem	3.09 (2.56–3.73)	2.97 (2.45–3.59)	3.01 (2.49–3.65)	2.71 (2.23–3.28)
Synergy index	0.90			
Housing tenure				
Owner, no minor mental health problem	1.00	1.00	1.00	1.00
Renter, no minor mental health problem	1.48 (1.24–1.76)	1.43 (1.19–1.71)	1.53 (1.28–1.83)	1.39 (1.16–1.66)
Owner, minor mental health problem	2.72 (2.28–3.24)	2.70 (2.26–3.22)	2.63 (2.20–3.14)	2.53 (2.12–3.02)
Renter, minor mental health problem	3.69 (2.98–4.57)	3.54 (2.85–4.40)	3.64 (2.94–4.52)	3.20 (2.57–3.99)
Synergy index	1.22			

The analyses were performed separately for each socioeconomic variable.

a Working conditions consisted of mental and physical strenuousness of work.

b Health behaviours consisted of smoking, problem drinking and leisure-time physical activity.

The joint associations of socioeconomic circumstances and minor mental health problems with antidepressant medication after adjusting for all socioeconomic circumstances simultaneously are in table 4 shows that the associations between childhood economic difficulties and low household income with antidepressant medication without minor mental health problems were abolished (table 4). Otherwise, the associations remained individuals with both minor mental health problems and childhood economic difficulties (OR 2.81, 95% CI 2.18–3.63), low household income (OR, 3.11, 95% CI 2.52–3.84), current economic difficulties (OR 2.98, 95% CI 2.43–3.66) or living in rented housing (OR 3.17, 95% CI 2.50–4.02) showing the strongest associations.

**Table 4:** The joint associations of socioeconomic circumstances and minor mental health problems with antidepressant medication (ORs and their 95% CIs)

	Gender, age=Model 1	**Model 1 + childhood economic difficulties, household income, current economic difficulties and housing tenure** ^a^
Childhood economic difficulties		
No, no minor mental health problem	1.00	1.00
Yes, no minor mental health problem	1.32 (1.03–1.68)	1.23 (0.96–1.57)
No, minor mental health problem	2.72 (2.30–3.21)	2.62 (2.22–3.10)
Yes, minor mental health problem	3.10 (2.41–3.99)	2.81 (2.18–3.63)
Household income		
High, no minor mental health problem	1.00	1.00
Low, no minor mental health problem	1.22 (1.02–1.46)	1.08 (0.90–1.31)
High, minor mental health problem	2.28 (1.83–2.84)	2.20 (1.76–2.74)
Low, minor mental health problem	3.62 (2.97–4.41)	3.11 (2.52–3.84)
Current economic difficulties		
No, no minor mental health problem	1.00	1.00
Yes, no minor mental health problem	1.43 (1.19–1.71)	1.29 (1.07–1.55)
No, minor mental health problem	2.88 (2.32–3.59)	2.86 (2.29–3.56)
Yes, minor mental health problem	3.38 (2.78–4.12)	2.98 (2.43–3.66)
Housing tenure		
Owner, no minor mental health problem	1.00	1.00
Renter, no minor mental health problem	1.41 (1.17–1.70)	1.26 (1.04–1.53)
Owner, minor mental health problem	2.69 (2.24–3.24)	2.57 (2.13–3.09)
Renter, minor mental health problem	3.74 (2.99–4.67)	3.17 (2.50–4.02)

a Each model was not adjusted for the socioeconomic variable also included in the joint model.

## Discussion

This study sought to examine the joint associations of socioeconomic circumstances and minor mental health problems with antidepressant medication. The significance of socioeconomic circumstances was small and minor mental health problems dominated the joint associations. Thus, employees with minor mental health problems showed strong associations with antidepressant medication largely irrespective of socioeconomic circumstances. Of the socioeconomic circumstances, material resources and current economic difficulties had the greatest contribution as they were associated with antidepressant medication even without minor mental health problems at baseline.

Our study underlines the importance of minor mental health problems to antidepressant medication irrespective of socioeconomic circumstances. Our results expand those of previous research as the outcome, antidepressant medication, portrays a medical diagnosis and seeking treatment. The results confirm previous findings showing that individuals with a risk of clinical depression might be recognizable by screening instruments^32^ and a further assessment by health care might be relevant. The incidence of antidepressant medication was rather high and early intervention might decrease human suffering and economic costs.

The synergy indices concerning the joint associations of minor mental health problems and household income and housing tenure were slightly synergistic suggesting that advantageous material socioeconomic circumstances might protect from minor mental health problems developing into more severe depression whereas the double burden of minor mental health problems and disadvantageous socioeconomic circumstances increases the risk. The finding should, however, be interpreted with caution as the confidence intervals partly overlapped. A previous study suggested that people in advantageous socioeconomic circumstances might be treated more often than people in lower socioeconomic circumstances despite similar needs for treatment^17^ and thus the results might underestimate the importance of socioeconomic circumstances. All in all, the results do not suggest major differences by socioeconomic circumstances in the course of minor mental health problems to major depression treated with antidepressant medication.

Among the socioeconomic circumstances our results emphasize the importance of material resources as household income and living in rented housing were associated with antidepressant medication even without minor mental health problems at baseline. Being better off financially might enable different alternatives both at work and in leisure time and provide freedom from financial restrictions. Also current economic difficulties were associated with antidepressant medication independent of minor mental health problems at baseline. These results confirm those of a previous study using the HHS data that examined the associations between multiple socioeconomic circumstances and antidepressant medication with a 5-year follow-up time.^18^ Also a previous study using the HHS data reported that the associations between conventional socioeconomic measures and minor mental health problems were weak and inconsistent whereas past and present economic difficulties increased the risk.^10^ A Swedish study found that low education was not associated with an increased risk of minor mental health problems whereas economic difficulties increased the risk.^12^ An Australian study reported that economic difficulties were strongly associated with depression above the effects of other socioeconomic circumstances.^33^ Economic difficulties are likely to reflect the influence of stress on depression.^34^ When adjusting simultaneously for all socioeconomic indicators the associations remained suggesting that childhood economic difficulties, household income, housing tenure and current economic difficulties are not interchangeable but each picture their own aspects of socioeconomic circumstances.

To shed light on the mechanisms behind the associations, the contribution of various covariates was examined. Interpersonal relationships have been found to prevent depressive symptoms.^35^ Albeit being an inadequate measure of interpersonal relations the associations were adjusted for marital status but no clear contribution was found. Also working conditions have been associated with depression.^36^ The associations were adjusted for both mental and physical work-load but they had no contribution. There are socioeconomic disparities in overweight^37^ and health behaviours,^38^ which have also been associated with depression.^39^^,^^40^ Adding these to the models minimally attenuated the associations. Our results thus suggest that these known determinants of depression only minimally contributed to the studied associations.

The strengths of the study include a relatively large dataset, a prospective study design and an opportunity to use a broad multi-domain approach to socioeconomic circumstances. The measure of depression used in the study, namely antidepressant medication was not optimal since it also measures treatment and not just incidence. On the one hand, it is a reliable register-based measure based on medical assessment without self-report bias and non-response. On the other hand, it is unlikely to detect all employees with diagnosed depression as part of the episodes of depression is treated by psychologies and psychotherapists. However, severe cases of depression are likely to be included as the current guidelines recommend medical therapy in such cases. Antidepressant medication is also used for other purposes, such as against chronic pain and anxiety disorders, but the most common indication is depression. We were unable to include a measure of depression not based on registers but a previous study found associations between socioeconomic circumstances and mental health using both self-reported measures of depression and antidepressant medication.^22^ Those who had purchased antidepressant medication for 3 years preceding the baseline were excluded as we wished to examine new cases. Depression is, however, recurrent by nature and some may have had earlier depression and the previous mental disorders may have influenced their employment participation and socioeconomic circumstances. Socioeconomic circumstances were dichotomized to secure a sufficient number of participants in each category when examining the joint association. This might obscure details of the associations by socioeconomic circumstances. The follow-up time was long and mental health problems and socioeconomic circumstances might have changed over time.

Of the original 8960 study participants 5450 were included in the study. The main exclusion was due to not consent to register linkage. According to the non-response analysis there were only small differences in consenting to the data linkages although men were more eager to consent. Another major reason for exclusion was due to omitting employees having purchased antidepressant medication 3 years preceding the baseline. Due to the exclusions employees in poorer physical and mental health might have selected out and the associations between minor mental health problems and antidepressant medication might be underestimates. The target population included only individuals employed at baseline and the most disadvantaged people suffering from mental health problems could not be covered. The associations might thus be stronger within the general population. The baseline data were collected in 2000–02 and studies with more recent data are needed to confirm whether the findings hold still in the present day.

Our findings stress the importance of minor mental health problems to antidepressant medication irrespective of socioeconomic circumstances. We also noted the importance of material resources and current economic difficulties as they were associated with antidepressant medication even without minor mental health problems at baseline. In conclusion, preventing minor mental health problems and less so, also improving socioeconomic circumstances might have protective influences against more severe depression.

## Supplementary data

Supplementary data are available at *EURPUB* online.

## Funding

A.S. was supported by the Emil Aaltonen Foundation. E.M. was supported by The Finnish Work Environment Fund (grant number 190256). O.R. was supported by the Juho Vainio Foundation.

##  

*Conflicts of interest*: None declared.

Key points

- Minor mental health problems dominated the joint association of past and present socioeconomic circumstances and minor mental health problems with antidepressant medication among midlife municipal employees.
- Of the socioeconomic circumstances, the study stressed the role of material resources and current economic difficulties as they were associated with antidepressant medication even without minor mental health problems at baseline.
- Preventing minor mental health problems and less so, also improving socioeconomic circumstances might have protective influences against more severe depression.

## Supplementary Material

ckac048_Supplementary_Data

## References

[1] Rehm J , Shield KD. Global burden of disease and the impact of mental and addictive disorders. Curr Psychiatry Rep 2019;21:10. 30729322 10.1007/s11920-019-0997-0

[2] Ferrari AJ , Charlson FJ, Norman RE, et al Burden of depressive disorders by country, sex, age, and year: findings from the Global Burden of Disease Study 2010. PLoS Med 2013;10:e1001547. 24223526 10.1371/journal.pmed.1001547 PMC3818162

[3] Pirkola S , Isometsä E, Suvisaari J, et al DSM-IV mood-, anxiety- and alcohol use disorders and their comorbidity in the Finnish general population. Results from the Health 2000 Study. Soc Psychiatry Psychiatr Epidemiol 2005;40:1–10. 15624068 10.1007/s00127-005-0848-7

[4] Markkula N , Suvisaari J, Saarni SI, et al Prevalence and correlates of major depressive disorder and dysthymia in an eleven-year follow-up–results from the Finnish Health 2011 Survey. J Affect Disord 2015;173:73–80. 25462399 10.1016/j.jad.2014.10.015

[5] Finnish Centre for Pensions, the Social Insurance Institution ofFinland, Official Statistics ofFinland, Social Protection 2019. NymanH, editor. Statistical Yearbook of Pensioners in Finland 2018. Helsinki: Finnish Centre for Pensions, 2019.

[6] Lorant V , Deliège D, Eaton W, et al Socioeconomic inequalities in depression: a meta-analysis. Am J Epidemiol 2003;157:98–112. 12522017 10.1093/aje/kwf182

[7] Wang JL , Schmitz N, Dewa CS. Socioeconomic status and the risk of major depression: the Canadian National Population Health Survey. J Epidemiol Community Health 2010;64:447–52. 19679710 10.1136/jech.2009.090910

[8] Jokela M , Keltikangas-Järvinen L. The association between low socioeconomic status and depressive symptoms depends on temperament and personality traits. Pers Individ Dif 2011;51:302–8.

[9] Schlax J , Jünger C, Beutel ME, et al Income and education predict elevated depressive symptoms in the general population: results from the Gutenberg health study. BMC Public Health 2019;19:430. 31014301 10.1186/s12889-019-6730-4 PMC6480596

[10] Lahelma E , Laaksonen M, Martikainen P, et al Multiple measures of socioeconomic circumstances and common mental disorders. Soc Sci Med 2006;63:1383–99. 16690186 10.1016/j.socscimed.2006.03.027

[11] Sekine M , Chandola T, Martikainen P, et al Socioeconomic inequalities in physical and mental functioning of British, Finnish, and Japanese civil servants: role of job demand, control, and work hours. Soc Sci Med 2009;69:1417–25. 19767137 10.1016/j.socscimed.2009.08.022 PMC2791858

[12] Molarius A , Granström F. Educational differences in psychological distress? Results from a population-based sample of men and women in Sweden in 2012. BMJ Open 2018;8:e021007. 10.1136/bmjopen-2017-021007 PMC5931303 29705766

[13] Goodwin L , Ben-Zion I, Fear NT, et al Are reports of psychological stress higher in occupational studies? A systematic review across occupational and population based studies. PLoS One 2013;8:e78693. 24223840 10.1371/journal.pone.0078693 PMC3817075

[14] Hays RD , Wells KB, Sherbourne CD, et al Functioning and well-being outcomes of patients with depression compared with chronic general medical illnesses. Arch Gen Psychiatry 1995;52:11–9. 7811158 10.1001/archpsyc.1995.03950130011002

[15] Silveira E , Taft C, Sundh V, et al Performance of the SF-36 Health Survey in screening for depressive and anxiety disorders in an elderly female Swedish population. Qual Life Res 2005;14:1263–74. 16047502 10.1007/s11136-004-7753-5

[16] Lundin A , Hallgren M, Theobald C, et al Validity of the 12-item version of the General Health Questionnaire in detecting depression in the general population. Public Health 2016;136:66–74. 27040911 10.1016/j.puhe.2016.03.005

[17] Kivimäki M , Gunnell D, Lawlor DA, et al Social inequalities in antidepressant treatment and mortality: a longitudinal register study. Psychol Med 2007;37:373–82. 17121686 10.1017/S0033291706009457

[18] Hansen DG , Søndergaard J, Vach W, et al Socio-economic inequalities in first-time use of antidepressants: a population-based study. Eur J Clin Pharmacol 2004;60:51–5. 14968270 10.1007/s00228-003-0723-y

[19] Mauramo E , Lallukka T, Laaksonen M, et al Past and present socioeconomic circumstances and psychotropic medication: a register-linkage study. J Epidemiol Community Health 2012;66:1143–51. 22523341 10.1136/jech-2011-200036

[20] Frandsen LS , Villumsen LB, Hjorth CF, et al The relationship between self-reported mental health and redeemed prescriptions of antidepressants: a register-based cohort study. BMC Psychiatry 2016;16:189. 27267897 10.1186/s12888-016-0893-7 PMC4897872

[21] Manescu EA , Robinson EJ, Henderson C. Attitudinal and demographic factors associated with seeking help and receiving antidepressant medication for symptoms of common mental disorder. BMC Psychiatry 2020;20:579. 33272233 10.1186/s12888-020-02971-9 PMC7711251

[22] Ahnquist J , Wamala SP. Economic hardships in adulthood and mental health in Sweden. The Swedish National Public Health Survey 2009. BMC Public Health 2011;11:788. 21989478 10.1186/1471-2458-11-788 PMC3206480

[23] Koskenvuo K , Koskenvuo M. Childhood adversities predict strongly the use of psychotropic drugs in adulthood: a population-based cohort study of 24 284 Finns. J Epidemiol Community Health 2015;69:354–60. 25538256 10.1136/jech-2014-204732

[24] Björkenstam E , Hjern A, Mittendorfer-Rutz E, et al Multi-exposure and clustering of adverse childhood experiences, socioeconomic differences and psychotropic medication in young adults. PLoS One 2013;8:e53551. 23341951 10.1371/journal.pone.0053551 PMC3547022

[25] Lahelma E , Aittomäki A, Laaksonen M, et al Cohort profile: the Helsinki Health Study. Int J Epidemiol 2013;42:722–30. 22467288 10.1093/ije/dys039

[26] City of Helsinki: Annual Report 2017. Helsinki Central Administration Publications 2018:16. Helsinki City Executive Office.

[27] Laaksonen M , Aittomäki A, Lallukka T, et al Register-based study among employees showed small nonparticipation bias in health surveys and check-ups. J Clin Epidemiol 2008;61:900–6. 18486445 10.1016/j.jclinepi.2007.09.010

[28] Hays RD , Morales LS. The RAND-36 measure of health-related quality of life. Ann Med 2001;33:350–7. 11491194 10.3109/07853890109002089

[29] WHO Collaborating Centre for Drug Statistics Methodology. Guidelines for ATC classification and DDD assignment. Oslo, 2013.

[30] Schofield A. The CAGE questionnaire and psychological health. Br J Addict 1988;83:761–4. 3255295 10.1111/j.1360-0443.1988.tb00508.x

[31] Andersson T , Alfredsson L, Källberg H, et al Calculating measures of biological interaction. Eur J Epidemiol 2005;20:575–9. 16119429 10.1007/s10654-005-7835-x

[32] Lundin A , Åhs J, Åsbring N, et al Discriminant validity of the 12-item version of the general health questionnaire in a Swedish case-control study. Nord J Psychiatry 2017;71:171–9. 27796153 10.1080/08039488.2016.1246608

[33] Butterworth P , Rodgers B, Windsor TD. Financial hardship, socio-economic position and depression: results from the PATH through life survey. Soc Sci Med 2009;69:229–37. 19501441 10.1016/j.socscimed.2009.05.008

[34] Pearlin LI , Schooler C. The structure of coping. J Health Social Behav 1978;19:2–21. 649936

[35] Molarius A , Berglund K, Eriksson C, et al Mental health symptoms in relation to socio-economic conditions and lifestyle factors -a population-based study in Sweden. BMC Public Health 2009;9:302. 19695085 10.1186/1471-2458-9-302 PMC2736164

[36] Theorell T , Hammarström A, Aronsson G, et al A systematic review including meta-analysis of work environment and depressive symptoms. BMC Public Health 2015;15:738. 26232123 10.1186/s12889-015-1954-4 PMC4522058

[37] Newton S , Braithwaite D, Akinyemiju TF. Socio-economic status over the life course and obesity: systematic review and meta-analysis. PLoS One 2017;12:e0177151. 28510579 10.1371/journal.pone.0177151 PMC5433719

[38] Pampel FC , Krueger PM, Denney JT. Socioeconomic disparities in health behaviors. Annu Rev Sociol 2010;36:349–70. 21909182 10.1146/annurev.soc.012809.102529 PMC3169799

[39] Jané-Llopis E , Matytsina I. Mental health and alcohol drugs and tobacco: a review of the comorbidity between mental disorders and the use of alcohol, tobacco and illicit drugs. Drug Alcohol Rev 2006;25:515–36. 17132571 10.1080/09595230600944461

[40] Luppino FS , de Wit LM, Bouvy PF, et al Overweight, obesity and depression. A systematic review and meta-analysis of longitudinal studies. Arch Gen Psychiatry 2010;67:220–9. 20194822 10.1001/archgenpsychiatry.2010.2

