# Supplementary material for: Joint association of socioeconomic circumstances and minor mental health problems with antidepressant medication
Source: Eur J Public Health. 2022 Jun 3;32(4):535–41. doi: 10.1093/eurpub/ckac048 (PMC9341681; doi:10.1093/eurpub/ckac048)
Supplement: Supplementary file 1 [file ckac048_supplementary_data.zip › ejph-2021-10-om-1006-File002.odt]

Supplemental Table S1. Distributions of joint association of socioeconomic circumstances and minor mental health problems (CMD) (n, %). Cumulative incidence of antidepressant medication by socioeconomic circumstances (%). 

|  | n | % | % with antidepressant medication during follow-up |
| --- | --- | --- | --- |
| Parental education |  |  |  |
| High, no minor mental health problem | 881 | 16 | 17 |
| Low, no minor mental health problem | 3098 | 57 | 16 |
| High, minor mental health problem | 363 | 7 | 33 |
| Low, minor mental health problem | 1074 | 20 | 34 |
| P value |  |  | <0.001 |
| Childhood economic difficulties |  |  |  |
| No, no minor mental health problem | 3208 | 63 | 15 |
| Yes, no minor mental health problem | 530 | 10 | 18 |
| No, minor mental health problem | 991 | 20 | 33 |
| Yes, minor mental health problem | 328 | 6 | 35 |
| P value |  |  | <0.001 |
| Education |  |  |  |
| High, no minor mental health problem | 2422 | 45 | 16 |
| Low, no minor mental health problem | 1559 | 29 | 16 |
| High, minor mental health problem | 923 | 17 | 33 |
| Low, minor mental health problem | 519 | 10 | 35 |
| P value |  |  | <0.001 |
| Occupational class |  |  |  |
| High, no minor mental health problem | 2087 | 38 | 16 |
| Low, no minor mental health problem | 1910 | 35 | 17 |
| High, minor mental health problem | 781 | 14 | 32 |
| Low, minor mental health problem | 667 | 12 | 36 |
| P value |  |  | <0.001 |
| Household income |  |  |  |
| High, no minor mental health problem | 2079 | 39 | 15 |
| Low, no minor mental health problem | 1842 | 34 | 17 |
| High, minor mental health problem | 656 | 12 | 29 |
| Low, minor mental health problem | 770 | 14 | 38 |
| P value |  |  | <0.001 |
| Current economic difficulties |  |  |  |
| No, no minor mental health problem | 2321 | 43 | 14 |
| Yes, no minor mental health problem | 1667 | 31 | 19 |
| No, minor mental health problem | 640 | 12 | 32 |
| Yes, minor mental health problem | 807 | 15 | 35 |
| P value |  |  | <0.001 |
| Housing tenure |  |  |  |
| Owner, no minor mental health problem | 2752 | 51 | 14 |
| Renter, no minor mental health problem | 1228 | 23 | 21 |
| Owner, minor mental health problem | 951 | 18 | 31 |
| Renter, minor mental health problem | 490 | 9 | 39 |
| P value |  |  | <0.001 |
